# Electrospun Nanofibrous Conduit Filled with a Collagen-Based Matrix (ColM) for Nerve Regeneration

**DOI:** 10.3390/molecules28227675

**Published:** 2023-11-20

**Authors:** Yuanjing Hou, Xinyu Wang, Yiyu Wang, Xia Chen, Benmei Wei, Juntao Zhang, Lian Zhu, Huizhi Kou, Wenyao Li, Haibo Wang

**Affiliations:** 1 School of Chemistry and Environmental Engineering, Wuhan Polytechnic University, Wuhan 430023, China; houyuanjing@whpu.edu.cn (Y.H.); benmeiwei@whpu.edu.cn (B.W.); zhangjt@whpu.edu.cn (J.Z.); yljzl@whpu.edu.cn (L.Z.); hzkou2007@163.com (H.K.); 2 State Key Laboratory of Advanced Technology for Materials Synthesis and Processing, Wuhan University of Technology, Wuhan 430070, China; 3 Institute of Nanobiomaterials and Immunology, School of Life Science, Taizhou University, Taizhou 318000, China; wangyiyu@tzc.edu.cn; 4 Sichuan Volcational College of Cultural Industries, Chengdu 610213, China; chenxia0810@163.com; 5 School of Materials Science and Engineering, Shanghai University of Engineering Science, Shanghai 200335, China; 6 College of Life Science and Technology, Hubei Key Laboratory of Quality Control of Characteristic Fruits and Vegetables, Hubei Engineering University, Xiaogan 432000, China

**Keywords:** biodegradability, electrospinning process, nanofibrous, porosity, Schwann cells

## Abstract

Traumatic nerve defects result in dysfunctions of sensory and motor nerves and are usually accompanied by pain. Nerve guidance conduits (NGCs) are widely applied to bridge large-gap nerve defects. However, few NGCs can truly replace autologous nerve grafts to achieve comprehensive neural regeneration and function recovery. Herein, a three-dimensional (3D) sponge-filled nanofibrous NGC (sf@NGC) resembling the structure of native peripheral nerves was developed. The conduit was fabricated by electrospinning a poly(L-lactide-co-glycolide) (PLGA) membrane, whereas the intraluminal filler was obtained by freeze-drying a collagen-based matrix (ColM) resembling the extracellular matrix. The effects of the electrospinning process and of the composition of ColM on the physicochemical performance of sf@NGC were investigated in detail. Furthermore, the biocompatibility of the PLGA sheath and ColM were evaluated. The continuous and homogeneous PLGA nanofiber membrane had high porosity and tensile strength. ColM was shown to exhibit an ECM-like architecture characterized by a multistage pore structure and a high porosity level of over 70%. The PLGA sheath and ColM were shown to possess stagewise degradability and good biocompatibility. In conclusion, sf@NGC may have a favorable potential for the treatment of nerve reconstruction.

## 1. Introduction

Peripheral nerve injury (PNI) is a clinically common degenerative disease with complex pathogenic mechanisms, which disrupts the signal transmission from the central nervous system to the limbs and body organs [1]. Once PNI occurs, patients may suffer from impaired sensation and reduced motor and autonomous function. Although PNI is usually accompanied by an active regeneration process, complete nerve regeneration and functional recovery have always been among the toughest clinical issues in neurosurgery, especially for long-segment defects [2]. Peripheral nerve regeneration consists of four main phases: establishment of a particular microenvironment and a channel suitable for axonal regrowth; axon sprouting and extension; regeneration of nerve fibers correctly in contact with the target organs; and myelination and maturation of the regenerated nerve [3]. It is a highly sophisticated pathophysiological process that involves various changes at different stages, concerning molecules, cells, and organs [4]. The therapeutic effect of treatments for nerve defect repair is closely associated with the extent of the injury and the strategy of nerve repair.

The current gold standard for repairing a long nerve segment is the implantation of an autograft to bridge the gap between two injured nerve stumps. However, nerve autografting also faces difficult challenges such as the scarce availability of donors, potential complications at the implantation donor site, compatibility issues between the donor and the recipient, as well as incomplete recovery [5,6]. As a result, the development of advanced nerve guidance conduits (NGCs) to replace autologous nerve transplantation has become an effective strategy. To date, it is established that an ideal NGC should provide adequate physical support, guide axon regeneration, avoid scar tissue infiltration and allow the diffusion of neurotrophic factors [7,8]. That is, specific microarchitectures and bioactive components should be considered when design and fabricating NGCs.

Over the past thirty years, substantial progress was achieved in developing autograft-like conduits utilizing different technologies, structures with a wide variety of geometrical arrangements, as well as various functionalized materials [9,10,11]. Notably, the electrospinning technique is widely utilized for nanofiber fabrication due to its ability to generate a hierarchical structure that mimics the extracellular matrix (ECM), featured with high specific surface area and porosity. According to a previous study, NGCs possessing precisely controlled nanofiber structures have the capability to offer contact guidance for the attachment and migration of Schwann cells (SCs), thereby facilitating the process of nerve regeneration [12]. Hollow conduits authorized by the U.S. Food and Drug Administration (FDA) are first-generation conduits used in clinical practice and include NeuroTube, Neurolac, NeuraGen, NeuroMatrix, etc. [3,8]. Nevertheless, these NGCs exhibit comparable efficacy to that of autologous transplantation only in repairing short-gap injuries (<2 cm) [13]. This is ascribed to wrong target innervation and axonal dispersion in the simple hollow conduits. Thus, various fillers like hydrogels, sponges, and filaments have been introduced in hollow tubes to resemble the endoneurial microarchitecture of native peripheral nerves. These inner fillers play a vital role in creating a desirable microenvironment for nerve regeneration, further directing axonal sprouting and extension [14,15].

In searching for effective approaches to successfully bridge nerve gaps, a range of natural and synthetic materials have been proposed. Collagen, a structural protein of the ECM, is present within the endoneurium and basal lamina and harbors cell binding motifs that aid in the promotion of axon outgrowth [16]. It has been extensively employed in the fabrication of NGCs to significantly improve the nerve regeneration process [17,18,19,20]. The observation of the reinnervation of a 10 mm sciatic nerve defect in rats revealed that a collagen–glycosaminoglycan conduit promoted a notable augmentation in axon number 6 weeks after implantation [21]. In fact, the application of pure collagen is hindered to a certain extent by undesirable features such as poor mechanical strength and fast degradation. Biodegradable aliphatic polyesters such as PLGA (a copolymer of glycolic acid (GA) and lactic acid (LA)) and its homologues have already been adapted for fabricating NGCs. A cylindrical nerve conduit made of PLGA (GA/LA = 10:90) exhibited favorable flexibility, biodegradability, permeability, and ease of suturing transected nerve stumps. When a PLGA NGC was surgically inserted into a 12 mm gap in the sciatic nerve of rats, a fibrin matrix filled the conduit, allowing for successful nerve regeneration [22]. PLGA exhibits desirable mechanical properties and processability, but hydrophobicity and lack of bioactive motifs limit its application. Evidence suggests that combining natural and synthetic polymers as well as using advanced fabrication techniques will allow obtaining favorable nerve grafts mimicking the microarchitecture of native nerves [23,24]. On the basis of the research results obtained so far, we fabricated a hybrid NGC consisting of a PLGA hollow tube and a collagen-based intraluminal filler, which is expected to promote the process of nerve regeneration thanks to the complementary functions of its components.

In this work, a hierarchical and biomimetic NGC (sf@NGC) composed of a nanofiber external tube and an inner filler was prepared by electrospinning and freeze-drying. The primary aim of this study was to develop sf@NGC utilizing a PLGA tube and a ColM intraluminal filler and evaluate its potential in nerve reconstruction therapy. We hypothesized that the unique properties of the PLGA sheath and ColM could enhance nerve regeneration and functional recovery. Specifically, ColM with an ECM-like topography was inserted into the PLGA nano-fibrous tube to construct a novel nerve-guiding conduit. Compared to other intraluminal fillers, ColM, with its randomly oriented fibers within the PLGA tube, offers both structural and biological support. This characteristic of ColM promotes cell alignment and guides axonal growth, facilitating nerve regeneration across the injured site. Additionally, the structural support offered by the PLGA tube would help prevent the collapse or compression of surrounding tissues, ensuring the integrity of the nerve pathway. This hybrid NGC with an ECM-like microstructure is believed to provide sufficient mechanical support and contact guidance for axons regrowth, further creating a desirable microenvironment for nerve regeneration.

## 2. Results and Discussion

### 2.1. Confirmation of the Successful Preparation of ColM

**Figure 1:**
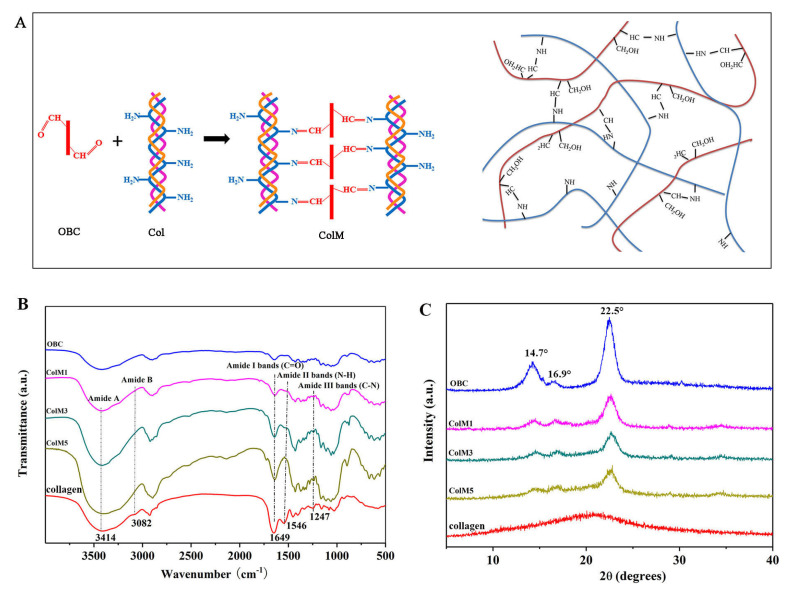
(**A**) Schematic formation mechanism of ColM by the crosslinking of Col and OBC, (**B**) FTIR spectra, and (**C**) XRD diffraction patterns of OBC, Col, and the series of ColM samples. Collagen is composed of three polypeptide chains that are intertwined with each other in a triple helix conformation. The three different colors represent the three polypeptide chains.

In order to imitate the natural architecture of peripheral nerves, 3D ColM samples consisting of collagen and OBC were prepared to be used as intraluminal fillers for NGCs. In our previous study, OBC emerged as a promising candidate for peripheral nerve repair due to its prominent mechanical properties, biocompatibility, and ECM-like structure [25]. It is worth to note that dialdehyde cellulose was reported to be an attractive crosslinker for modifying collagen due to its biocompatibility and eco-acceptability [26]. The combination of collagen with OBC is a facile strategy to enhance the inadequate mechanical strength and decrease the biodegradation rate of collagen. The reaction scheme for fabricating ColM is presented in Figure 1A. Amino groups like lysine, hydroxylysine, and arginine in collagen can bind the aldehyde groups of OBC through the Schiff base reaction.

FTIR. The FTIR spectra of Col, OBC, and the ColM fillers are presented in Figure 1B. The prominent absorption bands centered at 1740 cm^−1^ and 880 cm^−1^ were ascribed to the C=O stretching vibration and the formation of hemiacetal bonds in OBC [25]. In the spectra of the ColM samples, the characteristic peaks of OBC had nearly disappeared. Instead, three distinctive peaks occurred at 1649 cm^−1^, 1546 cm^−1^, and 1247 cm^−1^, which corresponded to amide I, amide II, and amide III, respectively [27]. The disappeared absorption bands and the new absorption peaks at 1649 cm^−1^ demonstrated the crosslinking reaction between Col and OBC. 

XRD. The typical XRD patterns of Col, OBC, and the ColM samples are reported in Figure 1C. Two distinct diffraction peaks appeared at 14.7° and 22.5° on the XRD pattern of OBC, which were ascribed to the crystalline fraction of cellulose [28], while a weak peak centered at 16.9° corresponded to the amorphous fraction of cellulose. In contrast, a broad peak present in the XRD pattern of pure collagen, indicated that collagen was amorphous. After crosslinking collagen with OBC, the ColM samples exhibited characteristic peaks corresponding to OBC. However, the intensities of the peaks corresponding to the crystalline fraction declined as the collagen ratio increased from 10% to 90%. The hindered crystallization of OBC was ascribed to the Schiff base reaction between amino groups in collagen and aldehyde groups in OBC. By combining these results with those of the FTIR analysis showing newly developed amide absorption peaks, these findings further validated the successful preparation of ColM.

**Figure 2:**
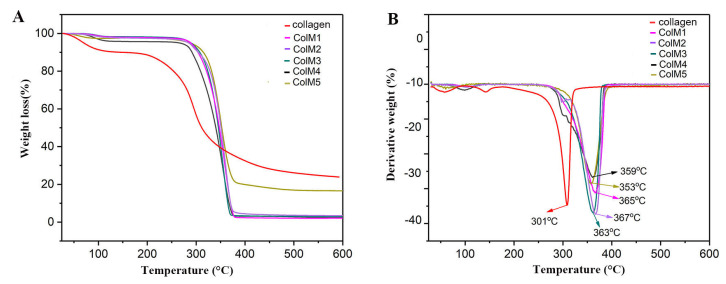
TGA (**A**) and DTG (**B**) thermograms of collagen and the series of ColM fillers with different proportions of collagen and OBC.

Thermal stability. Typical thermogravimetric (TGA) and differential thermogravimetric (DTG) curves of pure collagen and the series of ColM fillers are reported in Figure 2A,B. Two stages of weight loss were found in the TGA curves of all samples. The first weight loss measured from 50 °C to 120 °C was related to the breakage of hydrogen bonds as well as to the loss of small amounts of moisture. The subsequent reduction in weight occurred from 250 °C to 400 °C and corresponded to the thermal decomposition of each sample. Pure collagen showed a decomposition onset temperature (T_on_) of approximately 250 °C and a maximum decomposition temperature (T_max_) of 301 °C, whereas the T_on_ and T_max_ of ColM were significantly higher, reaching over 290 °C and 350 °C, respectively. In addition, the thermal stability of the COlM samples was improved as the crosslinking degree between collagen and OBC increased. The enhanced thermal stability of ColM can be attributed to the formation of Schiff base links between the aldehyde groups in OBC and the amino groups in the collagen molecule. The significant differences between the TGA curve of the ColM5 sample and those of the other ColM samples could indeed be due to the higher collagen-to-OBC weight ratio in ColM5. The 9:1 collagen-to-OBC ratio in ColM, indicating a greater amount of collagen in the composition of ColM5, could affect the thermal stability and degradation behavior of the sample, thus resulting in a different TGA curve. Collagen and OBC have different thermal properties; so, a high proportion of collagen could make the ColM5 sample behave differently under the same conditions compared to samples with lower collagen-to-OBC ratios.

**Figure 3:**
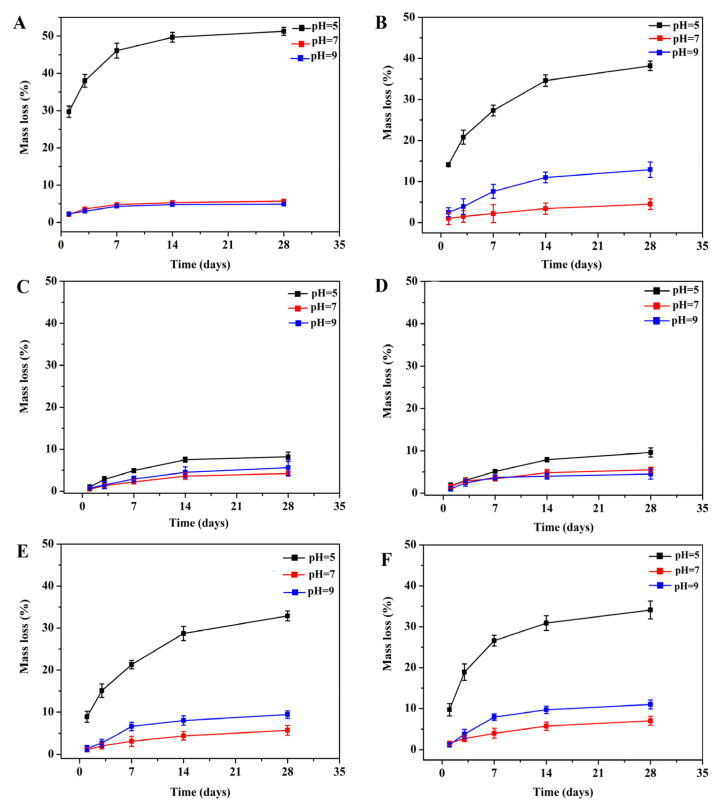
Mass loss of the ColM fillers incubated in three buffer solutions with different pH values. (**A**) collagen/BC composite, (**B**) ColM1, (**C**) ColM2, (**D**) ColM3, (**E**) ColM4, and (**F**) ColM5.

Solubility analysis. The solubility of implantable NGCs is crucial for the successful guiding of nerve regrowth and functional recovery. Therefore, the stability of the blended matrix was evaluated by investigating the weight loss of the ColM fillers in different buffer solutions. Figure 3 presents the mass loss of an un-crosslinked collagen/BC composite and various crosslinked ColM fillers after 28 days of incubation. All the ColM fillers exhibited good stability when exposed to neutral and alkaline environments. However, under acidic conditions, a noticeable mass loss was observed for the un-crosslinked collagen/BC composite and the ColM fillers with a lower degree of crosslinking (ColM1, ColM4, and ColM5). In contrast, the ColM2 and ColM3 fillers, possessing a higher crosslinking degree (Table S1, Supporting Information), displayed stability, with a mass loss below 10%. Therefore, a higher crosslinking degree provided the ColM fillers with increased stability and resistance to mass loss even in acidic environments. In fact, the microenvironment at the nerve injury site is typically acidic due to inadequate blood supply and presents inflammatory reactions caused by the metabolic byproducts of dead cells [29]. By maintaining stability in an acidic environment, ColM2 and ColM3 could support the attachment, growth, and migration of nerve cells, further facilitating the regeneration of an injured nerve.

### 2.2. Optimization of the Parameters of the Electrospinning Process

Electrospinning enables the easy production of micro/nanofibers that replicate the intricate structure of the native extracellular matrix (ECM). These fibers possess a high specific surface area, desirable pore interconnectivity, and customizable topography. Such characteristics are advantageous for promoting cell adhesion, proliferation, growth, and efficient nutrient delivery [30]. Numerous studies documented the use of scaffolds or matrices fabricated by electrospinning as viable options for tissue substitutes and drug delivery systems [30,31,32]. Electrospinning is an electrohydrodynamic process that involves charging a polymer solution or molten droplets to create a jet at the spinneret, which is subsequently followed by uniaxial stretching and elongation to generate fibers. Generally, an electrospinning set up involves a high-voltage power supply, a syringe pump, a needle spinneret, and a conducive collector [33,34]. It is known that the processing parameters have an great influence on the surface morphology and diameter of electrospun fibers. In this work, electrospinning was employed to fabricate a PLGA nanofiber membrane varying parameters such as solution concentration, spinning voltage, and liquid flow rate. A comprehensive investigation was conducted to examine the impact of these different parameters on the morphology and diameter of the obtained nanofibers. The average diameter (AD) value of the nanofibers was determined by analyzing SEM images with Image J (Image J 1.46r, National Institute of Health, Bethesda, MD, USA). In total, 100 fibers were randomly selected to measure their average diameter in each experimental condition.

**Figure 4:**
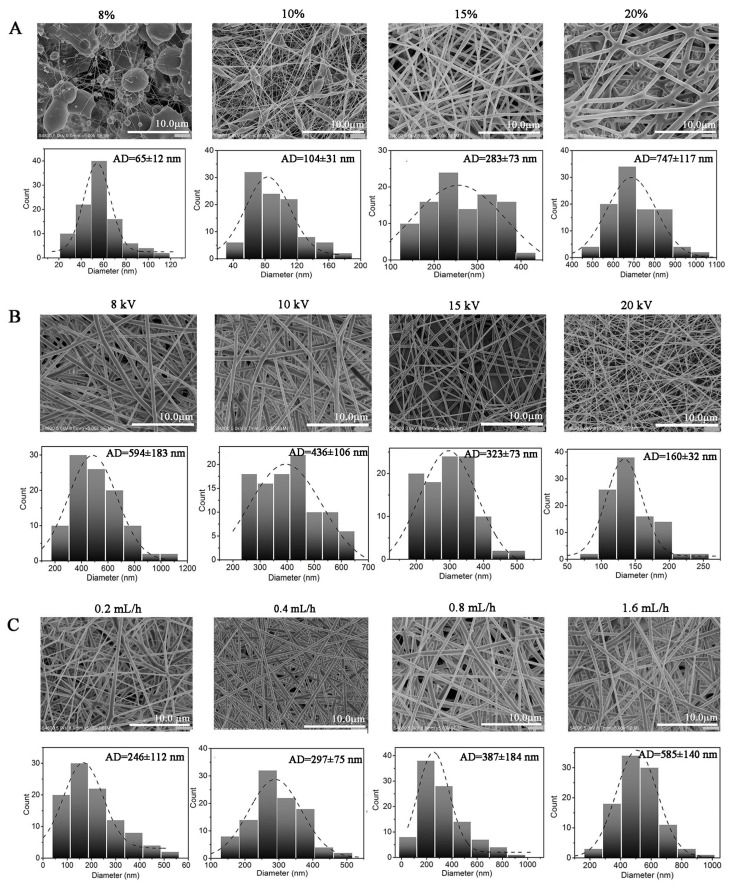
The SEM micrographs and the diameter distribution diagrams of electrospun PLGA fibers with different (**A**) concentrations of PLGA solution, (**B**) spinning voltages, and (**C**) liquid flow rate. The working distance between the spinneret needle and the collector was fixed at 15 cm during the experiments. Data are presented as means ± SD (*n* = 3).

PLGA concentration. To explore the impact of the initial PLGA concentration on the morphology of the nanofibers, solutions with PLGA concentrations varying from 8 to 20 wt% were prepared specifically for the electrospinning process. As shown in Figure 4A, at the low PLGA concentration of 8 wt%, only a limited number of nanofibers were observed, accompanied with a large number of beads. In this case, the low viscosity of the solution resulted in weak interactions among the polymer molecules, leading to the direct deposition or spraying of the liquid droplets onto the collector under the electric field, rather than to the stretching and elongation of the charged jet to form fibers. When the PLGA concentration was increased to 10 wt%, the resulting nanofibers exhibited a discontinuous and non-uniform structure, and fewer beads were observed. As the PLGA concentration increased to 15 wt%, bead-free, uniform, and consistent nanofibers were obtained. The gradual increase in the concentration of the PLGA solution promoted the formation of a Taylor cone, as well as the stretching, thinning, and solidification of the charged jet. Consequently, suitable nanofibers were collected. As the PLGA concentration was further raised to 20 wt%, the nanofibers exhibited aggregation and had a larger diameter. Moreover, the high viscosity of the solution resulted in the spinneret being prone to blockage, ultimately leading to a failure in the jetting process. An increase in the nanofiber AD from 65 ± 12 to 747 ± 117 nm was observed as the PLGA solution concentration increased from 8 wt% to 20 wt%. Taking into account these findings, the concentration of the PLGA solution was optimized to 15 wt%.

Spinning voltage. The spinning voltage directly determines the electrostatic force exerted on the liquid droplets, that is, the surface charge density of the jet. Fiber formation through spraying and elongation of the polymer jet occurs when the accumulated charge on the droplet’s surface surpasses the surface tension. In order to investigate the impact of applied voltage on fiber morphology, different spinning voltages ranging from 8 to 20 kV were applied for electrospinning, during which the PLGA concentration and liquid flow rate were kept constant at 15 wt% and 0.8 mL/h, respectively. As shown in Figure 4B, the AD of the nanofibers decreased from 594 ± 183 to 160 ± 32 nm, and the distribution range of the diameters became narrower with increasing spinning voltage. It is evident that higher voltage facilitated the formation of thinner fibers. However, as the voltage raised to 20 kV, a fluctuation of the jet was observed as a consequence of the intermittent flow of the PLGA solution, which was caused by the droplets being sprayed at a higher rate than the solution feeding rate. According to the findings of Yin et al. [35], a highly charged liquid jet is more prone to split into smaller jets, generating excrescent spots on the collector. This, in turn, hinders the achievement of fibers with a continuous, uniform, and smooth surface structure. It was reported that the highest voltage that can produce a continuous jet stream is named the quasi-stable point, at which a balance is achieved between the jet flow rate and the solution feeding rate [36]. Obviously, only with an appropriate increase in applied voltage, a continuous and uniform nanofiber will be generated from the spinneret. Thus, 15 kV was selected as the proper voltage for the electrospinning of PLGA.

Liquid flow rate. The liquid flow rate is also considered one of the vital parameters regulating the trajectory of the jet and the stability of the Taylor cone, further affecting the diameter and distribution of the fibers. The flow rate of the PLGA solution was varied from 0.2 to 1.6 mL/h to evaluate its impact on the morphology of the fibers. This investigation was carried out while maintaining a constant PLGA concentration of 15 wt% and a spinning voltage of 15 kV. A lower flow rate is generally advantageous for ensuring sufficient solvent evaporation, jet solidification, and proper deposition of the fibers. At a flow rate of 0.2 mL/h, only a small quantity of PLGA solution was expelled from the needle spinneret, and the liquid jet displayed intermittent breaks. By increasing the flow rate, the quantity of the ejected solution gradually increased, resulting in the formation of a stable jet and facilitating the production of continuous fibers. As shown in Figure 4C, the AD of the nanofibers increased from 246 ± 112 to 585 ± 150 nm with the increase in the flow rate from 0.2 to 1.6 mL/h. It is believed that lager droplets formed at higher flow rates. Consequently, the fibers were prone to merge together due to deficient stretching and elongation of the jet, as well as to an insufficient time for the residual solvent to dry. Therefore, the liquid flow rate was optimized at 0.4 mL/h to achieve continuous, uniform PLGA nanofibers with a smooth surface.

### 2.3. Characterization of the Electrospun PLGA Tube

**Figure 5:**
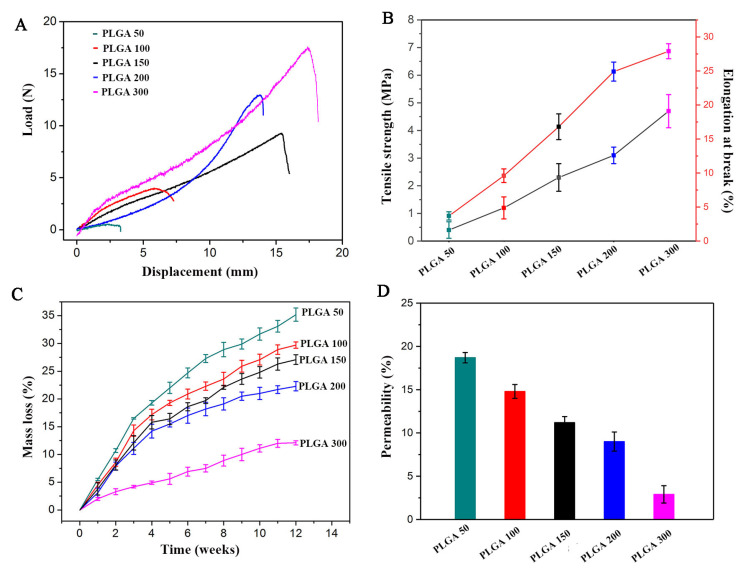
Mechanical, degradable, and permeable characterization of nanofibrous PLGA tubes. (**A**) Representative load–displacement curves, (**B**) tensile strength and elongation at break curves, (**C**) mass loss curves, and (**D**) permeability of a glucose solution in PLGA tubes with varying wall thickness. Data are presented as means ± SD (*n* = 3).

Mechanical properties. Desirable physicochemical features like adequate mechanical properties and appropriate biodegradability and permeability can be achieved in NGCs by adjusting their chemical composition and structure. The mechanical properties of NGCs are significantly influenced by factors such as structure, wall thickness, and porosity. In order to optimize the performance of the external conduit of sf@NGC, PLGA tubes with varying wall thicknesses of 50 μm (PLGA 50), 100 μm (PLGA 100), 150 μm (PLGA 150), 200 μm (PLGA 200), and 300 μm (PLGA 300) were prepared by adjusting the duration of the electrospinning process. As illustrated in Figure 5A, PLGA 50 was prone to break, with a quite small deformation. As the PLGA wall thickness increased from 100 to 300 μm, the tensile strength (TS) increased from 1.2 MPa to 4.7 MPa, while the elongation at break improved from 9.6% to 27.9% (see Figure 5B). NGCs need to exhibit adequate mechanical strength to ensure their structural integrity during surgical suturing, while also withstanding mechanical compression stresses from surrounding tissues until nerve regeneration is achieved. A study reported that the TS of the rat fresh sciatic nerve was 2.7 MPa [37]. Interestingly, both PLGA 200 and PLGA 300 exhibited comparable TS (3.1 MPa and 4.7 MPa, respectively) to that of the natural sciatic nerve.

In vitro degradation. A suitable degradation rate is crucial for implantable scaffolds; otherwise, complications may arise after long-term implantation. In general, the neurogenesis process takes approximately 2–3 months to complete. Studies on the therapeutic assessment of NGCs for long-gap nerve injuries may even last 3–48 months. FDA-approved commercial neural scaffolds such as Neuroflex and NeuroGen achieve a complete degradation after implantation for 8 and 48 months, respectively [38]. Figure 5C illustrates the in vitro degradation curves of PLGA tubes with different wall thickness. As expected, a significant mass loss was observed in all PLGA tubes after incubating them in PBS at 37 °C for 12 weeks. Among them, the PLGA 50 tube showed the highest mass loss, with a value of 35.2%. An excessively rapid degradation rate sacrifices the mechanical properties of NGCs, which is unfavorable for supporting nerve regrowth and extension. The degradation of PLGA primarily occurs due to the detachment of small molecules at the ends of molecular chains or side chains. As the wall thickness of the PLGA tubes increased from 100 μm to 300 μm, the mass loss rate gradually slowed down. PLGA 200 presented a relatively moderate mass loss. After 12 weeks of evaluation, the mass loss rate of PLGA 300 was only 12.1%. However, this slow degradation rate could inadvertently hinder nerve regeneration by limiting the space available for new nerve growth.

Permeability and porosity. Wall permeability is one of the vital characteristics of NGCs, as it enables the smooth transportation of nutrients and oxygen to cells. Studies indicated that the permeability of NGCs is affected by factors such as their thickness, degree of porosity, and pore size. Table 1 and Figure 5D show the porous characteristics and permeability of nanofibrous PLGA tubes with varying wall thickness. The PLGA 200 tube exhibited a porosity of 64.5 ± 2.3% and an average pore size of 11.8 ± 1.7 μm. There were no significant differences in porosity, average pore size, and pore size distribution between PLGA tubes with different thickness. It appeared that a pore size over 30 μm could result in the invasion of fibroblasts and in the loss of nutrients. This could be avoided with a pore size within the range of 10–20 μm [39].

To evaluate the impact of wall thickness on the permeability of PLGA tubes, the diffusion rate of the glucose solution was further examined. Following a 24 h incubation in PBS at 37 °C, PLGA 50 exhibited the highest permeability at 18.7%. At the same time, the permeability to the glucose solution decreased progressively from 14.8% to 11.2%, 9.6%, and then 2.9% for PLGA 100, PLGA 150, PLGA 200, and PLGA 300, respectively (Figure 5D). The decrease in permeability was attributed to the layer-by-layer stacking of the nanofibers, which impeded the diffusion of glucose. Therefore, considering the results of the analyses of the mechanical, degradation, and permeable properties, the optimal wall thickness for the PLGA tubes was determined to be 200 μm.

### 2.4. Morphology and Structure of sf@NGC

An ideal NGC should have certain physicochemical properties and be biocompatible; these features closely depend on the materials selected for NGC fabrication and on the structural design. Although hollow tubes contribute to promoting the nerve repair process, challenges such as nerve mismatch and incomplete functional recovery still persist. Introducing a specially structured matrix into hollow tubes to enhance axonal growth and extension is a promising strategy to achieve improved reinnervation. The natural ECM consists of randomly arranged fibers and plays a vital role in providing structural and biological support to tissues. Inspired by the natural architecture of peripheral nerves, we designed a biomimetic sf@NGC consisting of a tubular sheath and 3D sponge fillers.

**Figure 6:**
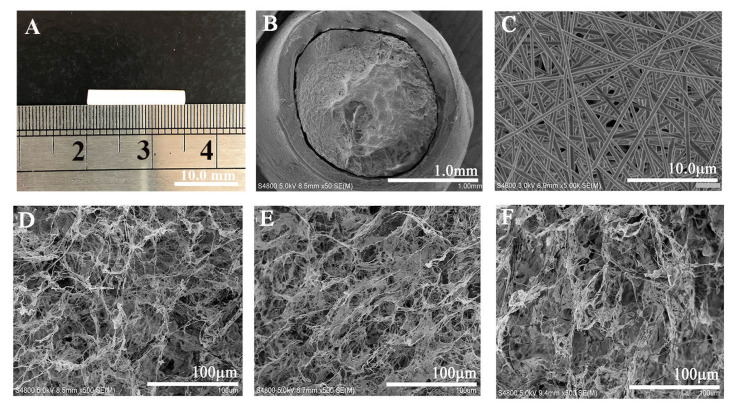
Photograph (**A**) and SEM characterization of sf@NGC. Transverse section of (**B**) sf@NGC showing a tight combination of the PLGA tube with ColM, (**C**) PLGA tube with uniform and smooth nanofibers, (**D**–**F**) ColM fillers with varying component proportions ((**D**), ColM1, (**E**), ColM3, and (**F**), ColM5).

The sf@NGC construct was produced by rolling the electrospun PLGA membrane into a tubular shape and subsequently inserting ColM into its inner lumen. Figure 6 shows a digital photograph and SEM images of sf@NGC. The data on the pore structure of ColM are summarized in Table 2. As shown in Figure 6B, the PLGA tube with an optimized wall thickness of 200 μm and a length of 15 mm was loaded with ColM2 fillers. The PLGA tube exhibited an external homogeneous and smooth nanofibrous composition, characterized by a porosity and a pore size of approximately 64.5 ± 2.3% and 11.8 ± 1.7 μm, respectively. The microscopic morphology of the ColM fillers is shown in Figure 6D–F. The ColM fillers possessed an ECM-like architecture with a 3D interconnected porous structure interwoven with high-aspect-ratio fibers. This biomimetic structure would facilitate the transport and exchange of nutrients and metabolites in the process of tissue regeneration. The porosity of the different ColM fillers was approximately 70%, while the average pore size slightly decreased with the increase in the proportion of collagen in the hybrid matrix. It was found that the ColM fillers presented 70% of medium pores (20–100 μm), 20% of large pores (>100 μm), and 10% of small pores (<20 μm). This multistage pore structure would be favorable for cell adhesion, proliferation, and migration, as well as support nutrient delivery and metabolite efflux [8].

### 2.5. Assessment of Biocompatibility In Vitro

**Figure 7:**
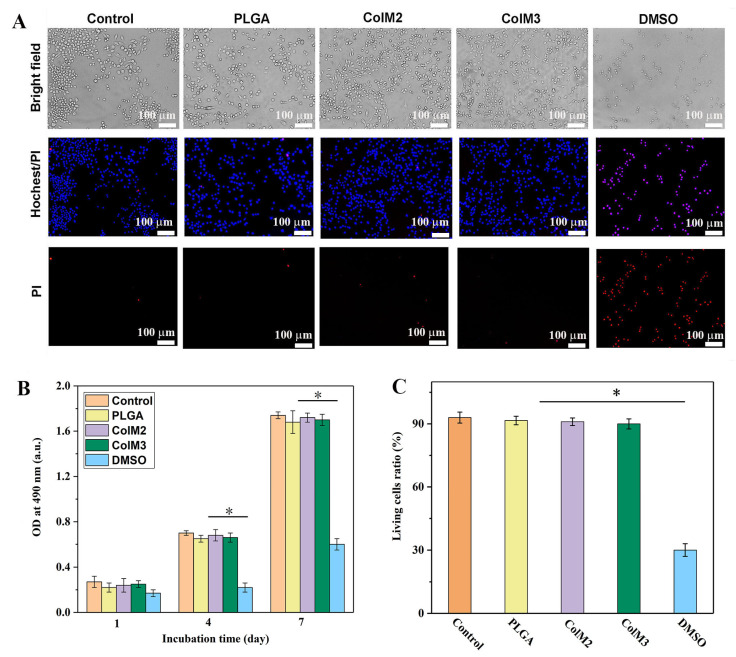
Assessment of the biocompatibility of the PLGA tube and ColM fillers in vitro using SCs. (**A**) Representative morphology of SCs cultured in media with extracts of the different samples for 2 days (SCs were stained with Hochest 33342 and PI fluorescent dyes), (**B**) cell viability assessment in different samples by the MTT assay, (**C**) statistical results of the survival rate of SCs cultured in different extracts for 2 days. Data are presented as means ± SD (*n* = 6, * *p* < 0.05).

SCs were used to assess the biological properties of the PLGA tube and ColM fillers in vitro using an extraction method. Figure 7 shows the results of morphological, cell viability, and living cell ratio analyses of SCs cultured in different conditioned media for different times. A Hoechst 33342 and PI staining kit was employed to determine the viability of SCs. Normal/apoptotic cells were dyed blue, and dead cells were dyed red. After 2 days of culture, comparable cell morphology and survival ratios were observed in the different conditioned media. Over 90% of SCs were found to be alive with abundant pseudopods. In contrast, a large number of dead SCs were discovered in the DMSO sample (Figure 7A,C). The viability of SCs cultured in different conditioned extracts was further determined by the MTT assay. Compared to the control group, no significant differences in OD were observed for SCs in PLGA, ColM2, and ColM3 conditioned media; significant differences were found for the DMSO group. These results showed that both PLGA and ColM fillers had no negative effects on cell viability and proliferation.

In the current study, we successfully developed a hybrid sf@NGC for nerve reconstruction therapy. The conduit was fabricated by electrospinning PLGA membranes to create a tube, while the internal filler was obtained by freeze-drying ColM, resulting in a structure that resembled the extracellular matrix. This development represents a significant step forward in the field of nerve reconstruction therapy. The study thoroughly investigated the impact of the electrospinning process and the composition of ColM on the physicochemical properties of sf@NGC. In addition, the biocompatibility of the PLGA sheath and ColM was assessed. The staged degradability and good biocompatibility of PLGA and ColM, along with the high porosity and tensile strength of the PLGA nanofiber membrane and the ECM-like structure of ColM, could potentially enhance nerve regeneration and functional recovery. While our study represents a progress in the field, further in vivo experiments are necessary to validate the effectiveness of sf@NGC in repairing long-gap nerve defects. This study provides a promising strategy for the treatment of peripheral nerve injuries, which are common but challenging clinical problems. sf@NGC could potentially improve the outcomes of nerve reconstruction surgery and reduce the associated morbidity. We believe that with constant research and development, sf@NGC could become a viable option for nerve reconstruction therapy in the near future.

## 3. Materials and Methods

### 3.1. Raw Materials

The copolymer poly(L-lactide-co-glycolide) (PLGA) (M_W_ = 100 kDa, LA/GA = 75/25) was offered by Jinan Daigang Biological Co., Ltd. (Jinan, China). Type I collagen was purchased from Sigma-Aldrich (St. Louis, MO, USA). Bacterial cellulose (BC) was obtained from Hainan Yida Food (Haikou, China). Dimethylacetamide, dichloromethane, and phosphate-buffered saline (PBS) were supplied by Sinopharm Chemical Regent Co., Ltd. (Shanghai, China). Fetal bovine serum (FBS), trypsin, and penicillin–streptomycin were purchased from HyClone (Logan, UT, USA). SCs were collected and cultured in our laboratory. 

### 3.2. Preparation of the 3D Collagen-Based Matrix (ColM)

Firstly, oxidized bacterial cellulose (OBC) was prepared by periodate oxidation of BC, as previously described [25]. Secondly, a well-dispersed OBC solution at a concentration of 0.1 mg/mL was prepared by ultrasonic emulsification. Thirdly, a 10 mg/mL collagen solution was mixed with the OBC solution at various mass ratios (1:9, 3:7, 5:5, 7:3, 9:1) and magnetically stirred for 24 h at 25 °C. Finally, the resulting mixtures were poured into cylindrical molds and then freeze-dried at −20 °C for 48 h to obtain the ColM. The crosslinking degree of the collagen and OBC mixtures is reported in Table S1.

### 3.3. Fabrication of the PLGA Tube and Optimization of the Parameters of the Electrospinning Process

PLGA particles were completely dissolved in a solvent mixture containing dimethylacetamide and dichloromethane (in a ratio of 1:3 *v*/*v*) at different concentrations, namely, 8 wt%, 10 wt%, 15 wt%, 20 wt%. The PLGA solutions were loaded into a syringe equipped with a needle (0.21 mm). Electrospinning was performed at various voltages (8–20 kV) and flow rates of the liquid (0.2–1.6 mL/h). The working distance between the needle and the collector was maintained constant at 15 cm during the experiments. The as-prepared PLGA nanofibrous membrane was dried overnight at room temperature to remove the residual solvent and then wrapped around a mold to obtain a tubular sheath. The thickness of the PLGA tube was measured using a hand-held digital thickness gauge at five randomly selected locations, and the average value was recorded.

### 3.4. Assembly of the Nanofibrous Sponge-Filled NGC (sf@NGC)

The ColM was gently inserted within the PLGA hollow tube to obtain sf@NGC. The fabrication process of sf@NGC is showed in detail in Figure S1. Afterwards, the as-prepared sf@NGC was sterilized for further use.

### 3.5. Characterization

#### 3.5.1. Fourier Transform Infrared (FTIR) Spectroscopy

The ColM samples were mixed with potassium bromide and compressed into pellets. FTIR spectroscopic measurements of the freeze-dried ColM samples were recorded on a Niccolet Avatar 360 spectrometer, performing 32 scans at a resolution of 1 cm^−1^. 

#### 3.5.2. X-ray Diffraction (XRD) Analysis

The XRD patterns of the lyophilized ColM samples were determined by a D/MAX-RB RU-200B diffractometer using a Cu Kα radiation source. Scanning was operated at 40 kV and 40 mA with 2 θ = 5–40°. The scanning rate and accumulation time were 2°/min and 15 s for per step, respectively.

#### 3.5.3. Thermal Properties

The thermal properties of pure collagen and the series of ColM samples were determined using a TGA Q500 calorimeter (TA Instrument, New Castle, DE, USA). Each sample prepared at same weight was scanned at a heating rate of 20 °C/min under a nitrogen flow of 25 mL/min. The thermogram curves were recorded between 25 °C to 600 °C for each sample. 

#### 3.5.4. Soluble Stability of the ColM Fillers

The ColM samples were incubated in three kinds of buffer solutions with pH values of 5 (acetate), 7 (PBS), and 9 (borate) at 37 °C. After incubating for 3, 7, 14, and 28 days, the samples were removed and thoroughly washed with distilled water, followed by freeze-drying to determine the mass loss. Each sample was weighed at least three times, and the average weight was recorded.

#### 3.5.5. Morphology Observation and Porosity Analysis

The microstructures of the PLGA nanofibrous tube and ColM fillers were examined by SEM (Hitachi S4800, Hitachi, Santa Clara, CA, USA). Before SEM observation, a thin layer of gold was applied to each sample as a coating. The porosity of the PLGA nanofibrous conduit and ColM fillers was measured by an automatic mercury porosimeter (AutoPore IV 9500, West Conshohocken, PA, USA) as previously described [25].

#### 3.5.6. Permeability Analysis

The electrospun PLGA tube was firstly incubated in a saline solution for 24 h. Then, 50 μL of a glucose solution was injected into the tube, which was sealed completely, followed by incubation in PBS at 37 °C for 24 h. The concentration of glucose in PBS after diffusion was measured by the GO kit, obtaining the percentage of glucose solution diffused through the wall into the outer PBS solution. As the concentration of the solution inside and outside the membrane was equal, the permeability of the fiber membrane was100%.

#### 3.5.7. Mechanical Property Analysis

The tensile strength of the PLGA nanofibrous membrane was acquired by a tension testing machine (Instron 5967, Instron, Norwood, MA, USA) equipped with a loaded cell of 100 N at 25 °C. Rectangular samples were uniformly prepared with length and width of about 50 and 10 mm. Before testing, the top and bottom of the samples were secured in place with fixtures mounted on the testing machine to prevent them from slipping or moving. A 30 mm long gauge was used during each tensile experiment.

#### 3.5.8. In Vitro Degradation Test 

The biodegradability of the PLGA nanofibrous tube was determined by incubating the samples in PBS for various times at 37 °C. At preselected time intervals, the samples were removed and extensively rinsed with distilled water, followed by freeze-drying and weighing. The weight loss ratio was acquired by Equation (1):

(1) $$Weight\;loss\;ratio\left(\%\right)=\frac{M_{0-}M_{t}}{M_{0}}\times 100\%$$

where M_0_ and M_t_ are the initial and post-degradation masses of the samples, respectively.

### 3.6. Biocompatibility Evaluation

SCs were cultured in complete DMEM containing 10% FBS and 1% penicillin/streptomycin at 37 °C in a 5% CO_2_ environment. The in vitro biocompatibility of the PLGA nanofibrous tube and ColM fillers was examined using an extraction method in accordance with the ISO 10993-5 standardized testing procedure [40]. Briefly, the samples were sterilized by immersion in ethanol 75% for 24 h followed by rinsing with PBS and then air-dried by irradiation under a UV lamp for 1 h. The sterilized samples were incubated in culture medium containing 10% FBS at 37 °C for 24 h, and extracts were acquired by filtering out the degraded particles. After 2 days of cultivation, SCs were dyed with Hoechst 33342 and PI using a double-fluorescence staining kit for 15 min and thoroughly washed with PBS. Then, the stained SCs were observed using a fluorescence microscope (IX71, Olympus, Tokyo, Japan). The living cell ratio was calculated by counting the number of alive and dead cells in the Hoechst 33342/PI staining acquired images using Image J 1.46r, National Institute of Health, USA (*n* = 50).

The viability of the cells was studied by a modified MTT [3-(4,5-dimethythiazol-2-yl)-2, 5 diphenyltetrazolium bromide assay. Specifically, SCs were seeded at a density of 1 × 10^3^ cells per well in 96-well culture plates and left adhere. After cellular adhesion, the culture medium was substituted with 100 μL of the extracts, and the cells were incubated at 37 °C in 5% CO_2_ for 1, 3, and 7 days. Subsequently, the extracts were removed, and the cells were incubated in 25 μL of MTT at 37 °C for 4 h. The reaction solution in each well was then substituted with 100 μL of DMSO to obtain the dissolution of blue formazan. Afterwards, the optical density (OD) at the wavelength of 490 nm was examined using a Bio-Tek (Winooski, VT, USA) Synergy HT microplate reader (*n* = 6).

### 3.7. Statistical Analysis

All data are expressed as means ± standard deviation (SD) of at least triplicate measurements. Statistical comparisons were evaluated using SPSS Statistics 24.0 software. Before conducting the statistical analysis, the data were tested for equal variances and normality. Values exhibiting unequal variances were subjected to log transformation and subsequently reassessed for equal variances. Differences between the groups were assessed for significance using one-way analysis of variance (ANOVA) followed by Tukey’s post hoc test. Differences were considered significant at *p* < 0.05.

## 4. Conclusions

In summary, the development of a biomimetic sf@NGC construct that mimics the architecture of native peripheral nerves was successfully achieved. sf@NGC consists of a PLGA tube and a ColM intraluminal filler. First, ColM was successfully developed through Schiff base linkage between amino groups in collagen and aldehyde groups in OBC, resulting in enhanced stability compared to pure collagen. Among all ColM specimens, ColM2 and ColM3 with crosslinking degrees of 6.8% and 5.7% showed optimal stability in acid environment. The ColM fillers exhibited a high porosity of 70% and an ECM-like architecture with an interconnected multistage pore structure. Second, homogenous and uniform PLGA nanofibers were obtained by optimizing solution concentration, spinning voltage, and liquid flow rate at 15 wt%, 15 kV, and 0.4 mL/h, respectively. The PLGA 200 tube exhibited considerable properties with a porosity of 64.5 ± 2.3%, a pore size of 11.8 ± 1.7 μm, and a tensile strength of 3.1 MPa. Third, studies conducted in vitro verified the biocompatibility of both the PLGA tube and ColM. This was demonstrated by the similar morphology and comparably high survival rates of SCs grown in the various conditioned media and control SCs and by the lack of significant differences in the OD value between treated SCs and control SCs. Overall, the combination of a PLGA nanofibrous conduit and ECM-like ColM fillers mimics the architecture of natural nerves and provides a supportive environment for cell growth, migration, and tissue regeneration. This research presents a novel approach for the treatment of large-gap nerve defects, aiming to achieve comprehensive innervation.

## Figures and Tables

**Table 1:** The porous properties of electrospun PLGA tubes with different thickness.

Samples	Porosity (%)	Average Pore Size (μm)	Pore Size Distribution (μm)
PLGA 50	58.2 ± 2.2	12.3 ± 1.3	4–18
PLGA 100	59.0 ± 1.0	10.5 ± 2.1	6–16
PLGA 150	62.8 ± 1.8	12.2 ± 1.6	2–25
PLGA 200	64.5 ± 2.3	11.8 ± 1.7	6–20
PLGA 300	64.8 ± 1.5	12.5 ± 2.4	5–22

**Table 2:** Characteristics of the pore structure of the ColM fillers quantified using mercury porosimetry.

Codes	Porosity (%)	Average Pore Size (μm)	Distribution of Pore (μm)				
			>100	100–20	20–10	10–5	<5
ColM1	72.3	60.2	23.3	72.4	3.4	0.2	0.7
ColM2	71.6	58.0	21.1	70.8	6.7	0.9	0.5
ColM3	70.1	57.4	19.4	70.5	8.8	0.6	0.7
ColM4	72.9	56.1	20.4	69.7	8.6	0.7	0.6
ColM5	71.8	55.0	18.8	71.7	7.2	1.0	1.3

## Data Availability

Data are contained within the article and Supplementary Materials.

## Supplementary Materials

The following supporting information can be downloaded at https://www.mdpi.com/article/10.3390/molecules28227675/s1, Figure S1: Schematic illustration of the fabrication of sf@NGC; Table S1: Sample codes and crosslinking degree of the ColM fillers. Reference [41] is cited in the Supplementary Materials.

## References

[1] Liu K., Yan L.S., Li R.T., Song Z.M., Ding J.X., Liu B., Chen X.S. (2022). 3D Printed Personalized Nerve Guide Conduits for Precision Repair of Peripheral Nerve Defects. Adv. Sci..

[2] Lu Q.Q., Zhang F., Cheng W.N., Gao X., Ding Z.Z., Zhang X.Y., Lu Q., Kaplan D.L. (2021). Nerve Guidance Conduits with Hierarchical Anisotropic Architecture for Peripheral Nerve Regeneration. Adv. Healthc. Mater..

[3] Yi S., Xu L., Gu X. (2019). Scaffolds for peripheral nerve repair and reconstruction. Exp. Neurol..

[4] Manoukian O.S., Rudraiah S., Arul M.R., Bartley J.M., Baker J.T., Yu X.J., Kumbar S.G. (2021). Biopolymer-nanotube nerve guidance conduit drug delivery for peripheral nerve regeneration: In vivo structural and functional assessment. Bioact. Mater..

[5] Spearman B.S., Desai V.H., Mobini S., McDermott M.D., Graham J.B., Otto K.J., Judy J.W., Schmidt C.E. (2018). Tissue-Engineered Peripheral Nerve Interfaces. Adv. Funct. Mater..

[6] Wieringa P.A., Gonçalves de Pinho A.R., Micera S., van Wezel R.J.A., Moroni L. (2018). Biomimetic Architectures for Peripheral Nerve Repair: A Review of Biofabrication Strategies. Adv. Healthc. Mater..

[7] Sarker M.D., Naghieh S., McInnes A.D., Schreyer D.J., Chen X. (2018). Regeneration of peripheral nerves by nerve guidance conduits: Influence of design, biopolymers, cells, growth factors, and physical stimuli. Prog. Neurobiol..

[8] Pinho A.C., Fonseca A.C., Serra A.C., Santos J.D., Coelho J.F.J. (2016). Peripheral Nerve Regeneration: Current Status and New Strategies Using Polymeric Materials. Adv. Healthc. Mater..

[9] Quan Q., Meng H., Chang B., Hong L., Li R., Liu G., Cheng X., Tang H., Liu P., Sun Y. (2019). Novel 3-D helix-flexible nerve guide conduits repair nerve defects. Biomaterials.

[10] Antman-Passig M., Giron J., Karni M., Motiei M., Schori H., Shefi O. (2021). Magnetic Assembly of a Multifunctional Guidance Conduit for Peripheral Nerve Repair. Adv. Funct. Mater..

[11] Wang J., Cheng Y., Wang H.Y., Wang Y.H., Zhang K.H., Fan C.Y., Wang H.J., Mo X.M. (2020). Biomimetic and hierarchical nerve conduits from multifunctional nanofibers for guided peripheral nerve regeneration. Acta Biomater..

[12] Brunelle A.R., Horner C.B., Low K., Ico G., Nam J. (2018). Electrospun thermosensitive hydrogel scaffold for enhanced chondrogenesis of human mesenchymal stem cells. Acta Biomater..

[13] Pateman C.J., Harding A.J., Glen A., Taylor C.S., Christmas C.R., Robinson P.P., Rimmer S., Boissonade F.M., Claeyssens F., Haycock J.W. (2015). Nerve guides manufactured from photocurable polymers to aid peripheral nerve repair. Biomaterials.

[14] Zou J.L., Liu S., Sun J.H., Yang W.H., Xu Y.W., Rao Z.L., Jiang B., Zhu Q.T., Liu X.L., Wu J.L. (2018). Peripheral Nerve-Derived Matrix Hydrogel Promotes Remyelination and Inhibits Synapse Formation. Adv. Funct. Mater..

[15] Huang L., Zhu L., Shi X., Xia B., Liu Z., Zhu S., Yang Y., Ma T., Cheng P., Luo K. (2018). A compound scaffold with uniform longitudinally oriented guidance cues and a porous sheath promotes peripheral nerve regeneration in vivo. Acta Biomater..

[16] Kehoe S., Zhang X.F., Boyd D. (2012). FDA approved guidance conduits and wraps for peripheral nerve injury: A review of materials and efficacy. Injury.

[17] Ma F., Xu F., Li R., Zheng Y., Wang F., Wei N., Zhong J., Tang Q., Zhu T., Wang Z. (2018). Sustained delivery of glial cell-derived neurotrophic factors in collagen conduits for facial nerve regeneration. Acta Biomater..

[18] Brown J.H., Das P., DiVito M.D., Ivancic D., Tan L.P., Wertheim J.A. (2018). Nanofibrous PLGA electrospun scaffolds modified with type I collagen influence hepatocyte function and support viability in vitro. Acta Biomater..

[19] Hoogenkamp H.R., Pot M.W., Hafmans T.G., Tiemessen D.M., Sun Y., Oosterwijk E., Feitz W.F., Daamen W.F., van Kuppevelt T.H. (2016). Scaffolds for whole organ tissue engineering: Construction and in vitro evaluation of a seamless, spherical and hollow collagen bladder construct with appendices. Acta Biomater..

[20] Bozkurt A., Boecker A., Tank J., Altinova H., Deumens R., Dabhi C., Tolba R., Weis J., Brook G.A., Pallua N. (2016). Efficient bridging of 20 mm rat sciatic nerve lesions with a longitudinally micro-structured collagen scaffold. Biomaterials.

[21] Deumens R., Bozkurt A., Meek M.F., Marcus M.A.E., Joosten E.A.J., Weis J., Brook G.A. (2010). Repairing injured peripheral nerves: Bridging the gap. Prog. Neurobiol..

[22] Faroni A., Mobasseri S.A., Kingham P.J., Reid A.J. (2015). Peripheral nerve regeneration: Experimental strategies and future perspectives. Adv. Drug Deliv. Rev..

[23] Tao J., Zhang J.M., Du T., Xu X., Deng X.M., Chen S.C., Liu J.L., Chen Y.W., Liu X., Xiong M.M. (2019). Rapid 3D printing of functional nanoparticle-enhanced conduits for effective nerve repair. Acta Biomater..

[24] Lu P., Wang G., Qian T., Cai X., Zhang P., Li M., Shen Y., Xue C., Wang H. (2021). The balanced microenvironment regulated by the degradants of appropriate PLGA scaffolds and chitosan conduit promotes peripheral nerve regeneration. Mater. Today Bio.

[25] Hou Y.J., Wang X.y., Yang J., Zhu R., Zhang Z.R., Li Y. (2018). Development and biocompatibility evaluation of biodegradable bacterial cellulose as a novel peripheral nerve scaffold. J. Biomed. Mater. Res. A.

[26] Adamiak K., Sionkowska A. (2020). Current methods of collagen cross-linking: Review. Int. J. Biol. Macromol..

[27] Li H., Cheng W., Liu K., Chen L., Huang Y., Wang X., Lv Z., He J., Li C. (2017). Reinforced collagen with oxidized microcrystalline cellulose shows improved hemostatic effects. Carbohydr. Polym..

[28] Klemm D., Heublein B., Fink H.P., Bohn A. (2005). Cellulose: Fascinating Biopolymer and Sustainable Raw Material. Angew. Chem. Int. Ed..

[29] Xi K., Gu Y., Tang J., Chen H., Xu Y., Wu L., Cai F., Deng L., Yang H., Shi Q. (2020). Microenvironment-responsive immunoregulatory electrospun fibers for promoting nerve function recovery. Nat. Commun..

[30] Ding J., Zhang J., Li J., Li D., Xiao C., Xiao H., Yang H., Zhuang X., Chen X. (2019). Electrospun polymer biomaterials. Prog. Polym. Sci..

[31] Collins M.N., Ren G., Young K., Pina S., Reis R.L., Oliveira J.M. (2021). Scaffold Fabrication Technologies and Structure/Function Properties in Bone Tissue Engineering. Adv. Funct. Mater..

[32] Xue J., Wu T., Dai Y., Xia Y. (2019). Electrospinning and Electrospun Nanofibers: Methods, Materials, and Applications. Chem. Rev..

[33] Rahmati M., Mills D.K., Urbanska A.M., Saeb M.R., Venugopal J.R., Ramakrishna S., Mozafari M., Mozafari M. (2021). Electrospinning for tissue engineering applications. Prog. Mater. Sci..

[34] Xing J.Y., Zhang M., Liu X.L., Wang C., Xu N.N., Xing D.M. (2023). Multi-material electrospinning: From methods to biomedical applications. Mater. Today Bio.

[35] Yin J.Y., Boaretti C., Lorenzetti A., Martucci A., Roso M., Modesti M. (2022). Effects of Solvent and Electrospinning Parameters on the Morphology and Piezoelectric Properties of PVDF Nanofibrous Membrane. Nanomaterials.

[36] Chen L.H., Ru C.B., Zhang H.G., Zhang Y.C., Wang H.X., Hu X.L., Li G. (2022). Progress in Electrohydrodynamic Atomization Preparation of Energetic Materials with Controlled Microstructures. Molecules.

[37] Borschel G.H., Kia K.F., Kuzon W.M., Dennis R.G. (2003). Mechanical properties of acellular peripheral nerve. J. Surg. Res..

[38] Yan Y., Yao R., Zhao J., Chen K., Duan L., Wang T., Zhang S., Guan J., Zheng Z., Wang X. (2022). Implantable nerve guidance conduits: Material combinations, multi-functional strategies and advanced engineering innovations. Bioact. Mater..

[39] Gu X.S., Ding F., Yang Y.M., Liu J. (2011). Construction of tissue engineered nerve grafts and their application in peripheral nerve regeneration. Prog. Neurobiol..

[40] (2009). Biological Evaluation of Medical Devices Part 5: Tests for In Vitro Cytotoxicity.

[41] Kim U.-J., Lee Y.R., Kang T.H., Choi J.W., Kimura S., Wada M. (2017). Protein adsorption of dialdehyde cellulose-crosslinked chitosan with high amino group contents. Carbohydr. Polym..

