# Supplementary material for: Electrospun Nanofibrous Conduit Filled with a Collagen-Based Matrix (ColM) for Nerve Regeneration
Source: Molecules. 2023 Nov 20;28(22):7675. doi: 10.3390/molecules28227675 (PMC10675555; doi:10.3390/molecules28227675)
Supplement: Supplementary file 1 [file molecules-28-07675-s001.zip › molecules-2678991-supplementary.pdf]

## Supporting Information

# Electrospun Nanofibrous Conduit Filled with a Collagen-Based Matrix (ColM) for Nerve Regeneration

**Yuanjing Hou [1], Xinyu Wang [2,*], Yiyu Wang [3], Xia Chen [4], Benmei Wei [1], Juntao Zhang [1], Lian Zhu [1], Huizhi Kou [1], Wenyao Li [5,*] and Haibo Wang [1,6,*]**

[1] School of Chemistry and Environmental Engineering, Wuhan Polytechnic University, Wuhan 430023, China; houyuanjing@whpu.edu.cn (Y.H.); benmeiwei@whpu.edu.cn (B.W.); zhangjt@whpu.edu.cn (J.Z.); yljzl@whpu.edu.cn (L.Z.); hzkou2007@163.com (H.K.)
[2] State Key Laboratory of Advanced Technology for Materials Synthesis and Processing, Wuhan University of Technology, Wuhan 430070, China
[3] Institute of Nanobiomaterials and Immunology, School of Life Science, Taizhou University, Taizhou 318000, China; wangyiyu@tzc.edu.cn
[4] Sichuan Volcational College of Cultural Industries, Chengdu 610213, China; chenxia0810@163.com
[5] School of Materials Science and Engineering, Shanghai University of Engineering Science, Shanghai 200335, China
[6] College of Life Science and Technology, Hubei Key Laboratory of Quality Control of Characteristic Fruits and Vegetables, Hubei Engineering University, Xiaogan 432000, China
* Correspondence: wangxinyu@whut.edu.cn (X.W.); liwenyao@sues.edu.cn (W.L.); wanghaibo@whpu.edu.cn (H.W.)

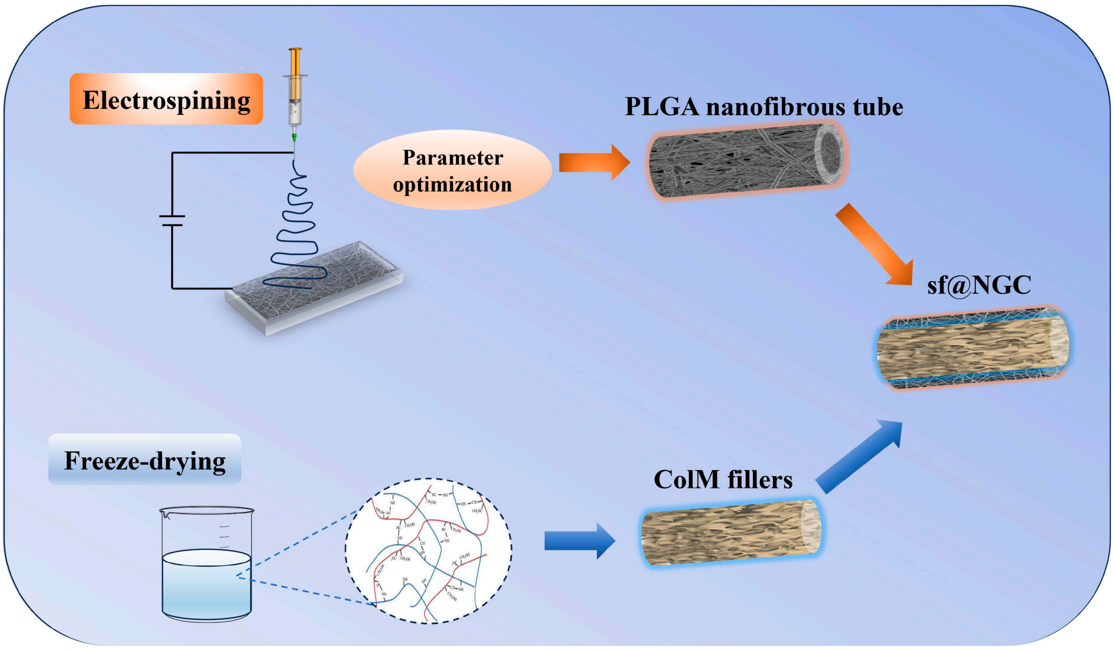


**Figure S1**. Schematic illustration of the fabrication course of sf@NGC.

Determination of crosslinking degree

The crosslinking degree for collagen and OBC was evaluated by an indirectly titration method as previously described [1]. In brief, ColM sample (100 mg) was dispersed in deionized water and 0.1 M HCl solution respectively, with stirring at room temperature for 4 h. And then 0.1 M NaOH was titrated to the suspension to achieve a neutral pH value. As free amine [$NH_2$] in collagen can protonate to amine [$NH^{3+}$] under the presence of HCl, the cross-linking degrees of ColM fillers were calculated by measuring the consumption of HCl.

Table S1 displays the crosslinking degrees of ColM fillers with different blend ratio of collagen and OBC. The crosslinking degree showed an initial increase and subsequent decrease with the rise in collagen content. Notably, ColM2 demonstrated the highest crosslinking degree, reaching a maximum of 6.8%. The lower crosslinking degrees observed in ColM1 and ColM5 fillers were likely a result of insufficient crosslinking reactions caused by imbalanced ratios of collagen and OBC.

**Table S1.** Sample codes and crosslinking degree of ColM fillers.

| Samples | Collagen: OBC mass ratio | Crosslinking degree (%) |
|---|---|---|
| ColM1 | 1:9 | 2.8 |
| ColM2 | 3:7 | 6.8 |
| ColM3 | 5:5 | 5.7 |
| ColM4 | 7:3 | 4.4 |
| ColM5 | 9:1 | 3.2 |

**Reference**

[1] U.-J. Kim, Y.R. Lee, T.H. Kang, J.W. Choi, S. Kimura, M. Wada, Protein adsorption of dialdehyde cellulose-crosslinked chitosan with high amino group contents, Carbohydrate Polymers 163 (2017) 34-42.
